# The general intervention model of policing: a narrative review of the literature

**DOI:** 10.3389/fpsyg.2025.1587452

**Published:** 2025-07-18

**Authors:** Stefan Schade, Markus M. Thielgen

**Affiliations:** ^1^Department Education and Research, Brandenburg State Police University, Oranienburg, Germany; ^2^Department Police, University of Applied Sciences for Police and Public Administration North Rhine-Westphalia, Aachen, Germany

**Keywords:** police operations, cognitive action model, knowledge, processing, action, mentally ill, psychosocial crisis, police interventions and mission management

## Abstract

Dealing with individuals suffering mental illness, being in psychological emergencies or crisis and any other exceptional circumstances is one of the most common professional requirements of police officers. Their personnel selection, education and advanced training must be consistently targeted toward successful coping with the real-world demands of police work. The question arises of what is necessary for police officers to be able to deal with individuals in psychological distress or exceptional situations without the use of force and the associated consequences on all those involved. In this article, we present a General Intervention Model of Policing (GIMP) consisting of three building blocks: (i) knowledge, (ii) processing, (iii) action. In this context, we propose that these empirically informed building blocks are necessary to achieve the aforementioned goal from the perspective of the police. We conceptualize the GIMP as a cognitive action model that particularly describes the mental processes of police officers involved. The model can be used as a basis for the development and empirical testing of a police requirement profile, as well as for the content of police recruitment and training. After presenting the building blocks of the model, it is placed in the context of other policing models and discussed.

## 1 Introduction

Dealing with people in emergency and crisis situations is part of the daily routine for police officers who have direct contact with the public (Livingston, 2016). For example, police officers may come into contact with individuals in mental health crisis situations during criminal interrogations or traffic controls. These encounters also include contact with individuals suffering from one or more mental disorders. Most of these contacts with mentally ill individuals are peaceful, without any use of force, which generally applies to all police-citizens-encounters in Germany. A mental disorder on its own is not usually a major issue in police operations. However, under certain circumstances, dealing with mentally ill persons can impose special demands on police officers, especially if there is a high risk to themselves and others. The following disorders are considered significant for the police: dementia, schizophrenia, affective disorders (depression, mania and bipolar disorder), personality disorders (especially antisocial or borderline personality disorder), mental disability and substance abuse, including alcohol intoxication (e.g., Biedermann and Ellrich, 2022; Hermanutz and Litzcke, 2004; Litzcke and Hermanutz, 2004; Wittmann, 2022; Posch, 2021; Wittmann and Posch, 2023). Given the prevalence of mental disorders in the population including certain police-related disorders such as depression (Jacobi et al., 2014) or reforms of the healthcare system and the associated tendency to de-institutionalize psychiatric patients (Salize et al., 2007), dealing with individuals in emergency mental health situations is one of the professional requirements for the police (Lorey and Fegert, 2021; Wittmann, 2022). On the other hand, people can get into emergency or exceptional situations even without suffering mental disorder and develop psychological symptoms (e.g., anxiety, panic, nervousness) that can lead to a problematic escalation within police operations. As a result, a wide range of behaviors with psychopathological or psychological symptoms are relevant to the police service. For various reasons and under certain conditions, the presence of the aforementioned disorders is associated with aggressive or violent behavior (Joyal et al., 2007). For example, it is estimated that a third of those shot dead by the police in Germany between 2007 and 2014 were probably mentally ill (Finzen, 2014; cf. Biedermann and Ellrich, 2022; Jasch, 2022). The figures, which are difficult to determine, can vary and may even be significantly higher (Bogner and Thurm, 2023; Fischhaber et al., 2022). In 2024, the number of individuals shot lethally by German police was the highest since the non-governmental organization ‘Bürgerrechte & Polizei/CILIP’ initiated its recording in 1976. 13 out of 22 individuals shot were described in press reports as suffering from mental illness or being in an exceptional psychosocial situation [CILIP (Bürgerrechte & Polizei/CILIP), 2025].

However, aggression and violence can also occur under situational (e.g., provocation, anger, frustration) or dispositional constellations (e.g., low self-control skills, high trait aggression, low trait anxiety) without interaction partners suffering from a mental disorder (Anderson and Bushman, 2002; Denson et al., 2012; DeWall et al., 2011; Finkel, 2014). The perceived threat and the resistance offered play a significant role in the use of police force and its intensity (Alpert and Dunham, 1997; Krajewski et al., 2023; McLean et al., 2023; McLean et al., 2019a; Stoughton et al., 2020; Terrill and Paoline, 2012). Although the aforementioned disorders are highly relevant in the police service, it is not the requirement or task of police officers on duty to carry out psychopathological diagnostics on those affected. It is undisputed that police officers are usually not psychologists or psychiatrists (Maier and Dittrich-Gessnitzer, 2023; Schmalzl, 2022). On the other hand, an undifferentiated and unreflected focus on lay “diagnoses,” such as “the crazy person,” “the zombie,” “the madman,” can also promote social categorization, stereotyping and stigmatization, which could lead to self-fulfilling prophecies by police officers and thus increase the risk of escalation.

Thus, the fundamental question arises as to how police officers should deal with individuals in mental emergencies and exceptional situations, regardless of their etiology, and how they should be prepared for them. In our opinion and in accordance with the literature, it is not the task of police officers to accurately diagnose clinical disorders. However, Maier and Dittrich-Gessnitzer (2023) state, that police officers should at least be able to recognize the mental states of their interaction partners in order to be able to react adequately to them. Presumably beyond the theory of mind (e.g., Brewer et al., 2018), the authors refer here to the recognition and understanding of mental states more specifically. In this context, Maier and Dittrich-Gessnitzer (2023, p.38) suggest the following “police-relevant mental states” on the basis of unstructured interviews with police officers from the North Rhine-Westphalian state police and the German federal police on “contact occasions with mentally ill persons and visible symptoms”: Delusions, hallucinations, hyperactivity, aggressiveness/impulsivity, anxiety, motor restlessness, despair. Even if they themselves attempt to justify a new “approach to dealing with the mentally ill in police practice,” the authors suggest that this remains an attempt at categorization with the risk of stigmatizing the interaction partners. It is therefore questionable whether this is a new approach or merely a shifting of the problem. An assessment of ‘behavioral abnormalities’ (Schmalzl, 2022) is similarly problematic and provokes a reductionistic dichotomization of ‘inconspicuous = normal’ versus ‘conspicuous = abnormal’ (cf. also Biedermann and Ellrich, 2022). Generally, it should be emphasized that the evaluation of individuals and their mental states by police officers on duty is obviously based on clinical, i.e., intuitive and subjective judgments without the use of standardized diagnostic instruments. Furthermore, it is noteworthy that these judgments may be carried out under conditions of stress. Consequently, they are susceptible to fallibility and bias (Kahneman, 2011).

In a democratic constitutional state, it is undisputed that the objective of policing concepts should be the safety of all those involved without any use of force. Police officers’ efforts must be directed toward this purpose (Manning, 2010). To this end, exceptional circumstances with the potential to endanger themselves and others must be identified, well-founded decisions must be asserted and an appropriate communicative response must be applied (Wolfe et al., 2020). The goal setting theory of motivation by Locke and Latham (1990, 2002) emphasizes the importance of goal setting for the regulation of action and the resulting performance. This does not fundamentally rule out the use of force. However, actions are orientated toward a non-violent solution to the situation if the goal is set accordingly. It can then be assumed that options for action will be chosen and efforts intensified that make a non-violent achievement more likely.

For the assessment and management of individuals in mental health emergencies and exceptional situations we propose a General Intervention Model of Policing (GIMP). Policing is a multi-faceted term and, in a wide conception, refers to the totality of all practices of social control and crime prevention that can be carried out by all social and political stakeholders including police organizations (Herrington and Roberts, 2021; Herrington and Colvin, 2016). In a closer conception of the term, policing refers to the police and their strategic activities as an organization, including the organizational culture or refers to the individual perceptions, thoughts and actions of police officers on duty (for more details on policing, see the early classics Banton, 1964; Bayley, 1994; Berkley, 1969; Bittner, 1970, 1990; Skolnick, 1966; see also the modern classics Bowling and Foster, 2002; Bowling et al., 2019; Dunham et al., 2021; Manning, 1988, 2003, 2018; Newburn, 2004, 2008, 2017; Reiner, 2010, 2015). In the present article, we primarily refer to the professional behaviors that police officers perform in the context of police-citizen encounters during police patrols to maintain law and order. Specifically, we focus on how police officers act cognitively and behaviorally when interacting with citizens. The distinctive characteristic of this interaction in a democratic constitutional state is the legal possibility of the use of force by the police. In the model, we disentangle the handling of interaction partners from the matter of illness or disorder. So, we consider police-citizens encounters in general, without emphasizing citizens under specific conditions. Instead, the model is applicable to various interaction partners in various situations and shifts the conceptual focus to the importance of police officers’ self-regulatory skills and individual competencies. Irrespective of the categorization of the mental state and its labeling, we argue that education and training of police officers should obviously be based on their competence profile (Bennell et al., 2022; Wolfe et al., 2020). In motivation psychology, White (1959, p. 317) describes competence as ‘effective interaction (of the individual) with the environment’. In psychology and educational research, Weinert’s definition (Weinert, 2001) has become established. Accordingly, competencies are “the cognitive abilities and skills that individuals possess or can learn in order to solve certain problems, as well as the associated motivational, volitional and social willingness and ability to appy the problem solutions successfully and responsibly in variable situations” [„die bei Individuen verfügbaren oder durch sie erlernbaren kognitiven Fähigkeiten und Fertigkeiten, um bestimmte Probleme zu lösen sowie die damit verbundenen motivationalen, volitionalen und sozialen Bereitschaften und Fähigkeiten, um die Problemlösungen in variablen Situationen erfolgreich und verantwortungsvoll nutzen zu können“] (Weinert, 2001, p. 27 ff.; cf. also Weinert, 1999, 2001)”. In this understanding, competence is a disposition that enables people to successfully solve certain types of problems, i.e., to cope with specific challenging situations of a certain type’ [„Kompetenz ist nach diesem Verständnis eine Disposition, die Personen befähigt, bestimmte Arten von Problemen erfolgreich zu lösen, also konkrete Anforderungssituationen eines bestimmten Typs zu bewältigen“] (Klieme et al., 2003, p. 72). Thus, especially in professional and educational contexts, the concept of competence is distinguished from the concept of a rather crystallized intelligence (Cattell, 1963), which describes cross-domain, relatively time-stable and hardly changeable cognitive abilities and refers to performance in a specific practical context (Hartig and Klieme, 2006, p. 135; McClelland, 1973). Within a professional context, (professional) action competence refers to the ability to successfully master complex professional requirements. This context-bound ability must be distinguished from performance as actually observed success. Competencies are not merely context-bound, but are also subject to change due to learning processes. Therefore, they are target of education and training (Wolfe et al., 2020; cf. also Hartig and Klieme, 2006; Klafki, 1964; Klieme, 2004; Klieme et al., 2003, pp. 72–73; Klieme and Leutner, 2006, p. 879; Klieme and Hartig, 2007; Roth, 1971). Thus, police competence refers to the successful mastery of operational requirements, which can relate to different operational contexts, e.g., investigative interviewing, traffic controls, patrols, etc. (Schade, 2022).

**Figure 1:**
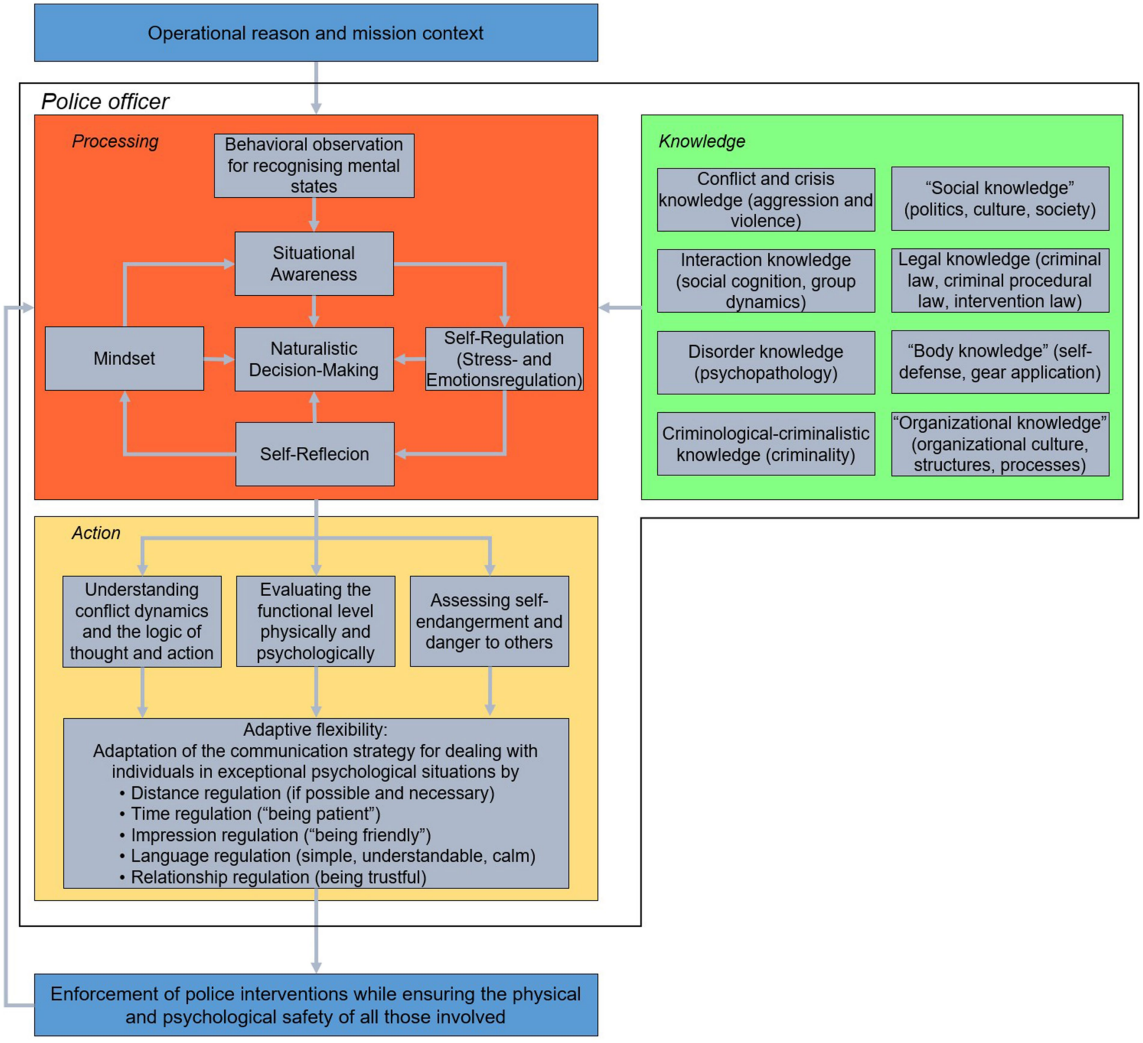
Architecture of the general intervention model of policing and its components “knowledge,” “processing” and “action.” The model specifies these components as a prerequisite for the successful management of complex and dynamic police operations (Source: Authors illustration).

The initial point of police action is the reason for the operation and the operational context. Within the special context of police operations police officers need to solve practical problems. Therefore, police officers professional action competence is required to successfully cope with complex demands of police operations. The present model displayed in Figure 1 consists of the three building blocks: (i) knowledge, (ii) processing, (iii) action. The interplay of this building blocks determines the successful mastery of police demands. As mentioned above, the conceptual focus lies on police officers’ self-regulatory competencies. Therefore, the building block of processing plays a prominent role within the model. The knowledge components form the theoretical foundation for police action including all domains of knowledge that we consider necessary for coping with police requirements. The processing components essentially relate to the mental processes (cognitions) of police officers. Therefore, we understand the present model as a cognitive action model. As key processes, we identify situation awareness including naturalistic decision-making, self-control and self-regulatory competence including stress and emotion management as well as the police mindset in addition to self-reflection. As already mentioned, professional contexts require context-specific performances to cope with practical requirements. The action component of the GIMP relates to specific actions of police officers operating in the field. Based on an understanding of the specific situational dynamics, including their personal thinking and action logic as well as that of their interaction partners in addition to an assessment of the physical and psychological level of functioning and the risk constellations derived from this, we propose structuring situational communication behavior with the help of five action dimensions: (a) impression regulation, (b) distance regulation, (c) time regulation, (d) language regulation, (e) relationship regulation. The interaction of these five regulatory dimensions should enable flexible adaptation to the interaction partners. The general objective of police is to carry out police interventions without the use of force. Insights into how crisis situations can be resolved peacefully come from modern research on hostage negotiation (Grubb, 2010, 2023). Recently, several models of crisis negotiation have been proposed: BISM (Vecchi et al., 2005), S. A. F. E. model (Hammer and Rogan, 1997), STEPS model (Kelln and McMurtry, 2007), D. I. A. M. O. N. D. model (Grubb et al., 2020). For example, according to the framework of the Behavioral Influence Stairway Model (BISM, Ireland and Vecchi, 2009; Vecchi et al., 2005, 2019) active listening skills are used to initiate a relationship-building process between the police officer and the opponent in both barricaded hostage and crisis situations. Based on the development of rapport, individuals can be encouraged toward a peaceful resolution of the critical incident.

The GIMP can be used for developing and empirically testing police job profiles and for configuring the content of both police personnel selection procedures and basic or advanced training sessions. Since police work involves successfully coping with professional demands in a specific context, the actions of the police officers cannot be considered independently of the specific operational context. After presenting the building blocks of the model, it is discussed in the context of other police operational models. Before the three main components are presented, we characterize the special nature of the context of police operations.

## 2 The present study

In the present study, a narrative literature review was conducted to answer the research question of how police officers generally act cognitively during police operations. This qualitative method enables a comprehensive and critical examination of the existing literature on the research question without claiming to be an exhaustive systematic review of the literature (Baumeister and Leary, 1997; Ferrari, 2015; Green et al., 2006). The objective of the present article is to identify central concepts and current research findings on the cognitive performance of police officers in the field and to integrate them into a conceptual framework. The selection of literature is based on its relevance to the research question and its theoretical or empirical significance. Instead, the focus is on a selective, contextualized and interpretative presentation of the relevant literature, which makes the selection of literature subjective and therefore possibly biased. However, the narrative literature review can provide the foundation for a structured description of the current state of research for further analysis in order to develop or to clarify theoretical concepts and to identify research gaps. Especially in an interdisciplinary application context such as the police, the narrative literature review can help to organize relevant literature. German and English-language publications were included in this narrative literature review.

## 3 Building blocks

### 3.1 Operational reason and mission context

Police officers are encountered with various people under different conditions during their deployments. These so-called zero acquaintances are dynamic interactions between the individuals involved (Thielgen and Schade, 2023). Consequently, on an individual level, mutual impression management and personal judgment, including cognitive biases in information processing, play a central role (Staller et al., 2023a; Zaiser et al., 2023). In addition, group phenomena are also central, as police operations are rarely bilateral and group memberships and social identity processes are permanently salient (Bradford et al., 2014; Bradford, 2012; Filstad, 2022; Hazen and Brank, 2023; Hoggett et al., 2019; Loftus, 2009; Murphy et al., 2018). Police-citizen encounters can occur for various reasons (e.g., accident, brawl, suspected crime, routine check, etc.) depending on the context (criminal investigation, patrol, traffic control, demonstration, etc.). The potential for conflict and violence can arise when the goals and interests of citizens and police officers differ, which usually lies in the nature of the encounter (Koerner and Staller, 2022a). Individuals can find themselves in an exceptional or emergency situation for a wide variety of reasons. In around 20% of police operations, individuals suffering from mental illness are at the center of the incident (Lorey and Fegert, 2021). Suffering from a mental disorder can exacerbate a crisis situation. When certain risk constellations occur cumulatively (e.g., mental disorder, alcohol abuse, low self-control, antisocial attitudes, etc.), violence becomes more likely (Anderson and Bushman, 2002; Bonta and Andrews, 2023; Finkel and Hall, 2018; Slotter and Finkel, 2011).

The information processing of both police officers and citizens is influenced by various internal and external factors (e.g., Thielgen and Schade, 2023). The appraisal of the danger significantly affects the assessment of the situation on both sides. As a result, experiencing fear makes impulsive actions, such as fight or flight, more likely. Police officers are likely to increase their efforts to protect themselves, which may lead to the use of force (Koerner et al., 2023b; Staller et al., 2023b). Citizens could feel additionally threatened by this and resist accordingly. All involved in police operations can be particularly affected by the stress they experience. The information processing, action planning and execution of both police officers and citizens is usually impaired by the effects of stress (Anderson et al., 2019; Colligan and Higgins, 2006; Hine et al., 2018; Jäger and Kohls, 2023; Kelley et al., 2019; Schade and Schack, 2023). In addition to operational stress, police officers can also experience organizational stress in the form of workload and its long-term chronic effects, which increases the overall stress level (Frenkel and Uhlenbrock, 2023; Gutschmidt and Monecke, 2023). As well as police officers, citizens can also be exposed to other stress factors arising from their social and professional lives (e.g., partnership conflicts), which can intensify the experience of stress during police operations. As a result, both police officers and citizens may be exposed to considerable amounts of stress during a police operation, which usually impairs their cognitive and behavioral functioning.

The organizational context of the police can also determine police action. Traditionally, a distinction is made in the police between a formal and an informal part of the organizational culture (Behr, 2006, 2008; Behr, 2000; Ianni and Ianni, 1972; Reiner, 2002; Reiner, 2000; Reuss-Ianni, 1983). The official police culture provides the constitutional basis for police action through laws, ordinances, decrees and service regulations. In contrast, there is the informal cop culture as “‘concentrate’ of everyday police knowledge” [„, Konzentrat‘des polizeilichen Alltagswissens“] (Behr, 2006, p. 39), which provides experience-based instructions for police officers that helps to transform official guidelines into concrete operational situations. Under the influence of cop culture, police officers can see themselves as a “community of danger.” The associated loyalty to one another can lead to an esprit de corps that results in a code of silence in the case of the illegal use of force, that is even opposed by the principle of legality as a rule of law (Behr, 2009; Chin and Wells, 1998; Rothwell and Baldwin, 2007; Skolnick, 2002; Westmarland, 2005). As representatives of the executive power of the constitutional state, police officers are authorized to use force legally within certain restrictions. This role makes the interaction between the police and the public asymmetrical. Therefore, authority and dominance can determine the interactions in police operations (Alpert et al., 2004; Koerner et al., 2023a; vom Hau, 2022). From the citizens’ perspective, the extent to which police actions are perceived as procedurally fair and trust can be built in the police plays a central role in this context (Staubli, 2022; Tyler, 2001).

Although the conditions of police operations can be specified, e.g., as risk factors for the use of force, the outcome of an operation cannot be predicted linearly (deterministically) from the actions of the individual participants or their interactions. Rather, individual incidents are more or less likely in the sense of the concept of conditional probabilities if certain factors are present. An accumulation of (risk) factors can increase the probability of incidents’ occurrence, which does not mean that its occurrence is guaranteed (Staller et al., 2023c; Staller et al., 2022a). Even if the probability of the integrity and safety of all those involved increases, in a complex and dynamic operational situation influencing factors can become apparent that cause the opposite to occur. In summary, police operations can be characterized as follows: complex instead of simple, dynamic instead of static, non-linear instead of linear (or probabilistic instead of deterministic). Therefore, police officers are called upon to use their skills to satisfy these requirements.

### 3.2 Knowledge

The knowledge components form the theoretical foundation for policing. This includes all areas of knowledge that the authors consider necessary for coping with police requirements. Practically, empirical informed job profiles are required. In the context of professional aptitude diagnostics, a requirements analysis, for example with the help of the critical incident technique by Flanagan (1954), is first used to identify the elements of a job that are critical to success and then to derive personal characteristics, usually summarized as KSAOs (knowledge, skills, abilities and other characteristics), which are required to successfully perform the job (Boyatzis, 2008). Bennell et al. (2022) reviewed the literature revealing 10 such KSAOs that are frequently discussed as underlying policing. These KSAOs comprise: “(1) knowledge of policies and laws; (2) an understanding of mental health-related issues; (3) an ability to interact effectively with, and show respect for, individuals from diverse community groups; (4) awareness and management of stress effects; (5) communication skills; (6) decision-making and problem-solving skills; (7) perceptual skills; (8) motor skills related to use-of-force; (9) emotion and behavior regulation; and (10) an ability to treat people in a procedurally just manner” (Bennell et al., 2022, p.1). For the first time in Germany, Nettelnstroth et al. (2019, 2020) were able to generate a police job profile using the person-related empirical method that linked police officers’ individual characteristics and job success measures within the Hamburg state police to derive empirically based characteristics that underlie the police profession. These include the following broad and relatively time-stable categories: (1) “reflective attitude” (i.e., “appropriate” behavior on the job, learning from criticism, sense of responsibility, identification with the police), (2) “commitment and interest” (i.e., pronounced willingness to work and perform, high job satisfaction and personal initiative), (3) “social competence and cooperation” (i.e., working together in a team, communicating “appropriately,” having a positive influence on working relationships through open-mindedness and collegiality), (4) “mental stability” (i.e., “healthy” self-confidence and trust in one’s own abilities, good handling of high stress levels, balanced emotional reactions) and (5) “cognitive skills” (i.e., having practical specialist knowledge). A more generic approach is the great eight competence model by Bartram (2005). This model is an empirically based competency model for describing job-relevant competency domains and corresponding behaviors, that are associated with successful professional performance. It is based on a comprehensive meta-analysis of scientific studies on existing competency models and combines 112 individual competencies as precisely defined and observable behaviors into 20 overall competency domains, which in turn are assigned to eight competency clusters. These eight competency clusters are: (1) leading and deciding, (2) supporting and cooperating, (3) interacting and presenting, (4) analyzing and interpreting, (5) creating and conceptualizing, (6) Organizing and executing, (7) adapting and coping, (8) enterprising and performing. Due to its behavioral and work-related operationalization, the model is used in personnel selection and development at both operational and leadership level. As far as we know, it has not yet been applied to the police context (see also Campion et al., 2011).

Since policing requires interpersonal interaction both internally among team members and externally with citizens, social-psychological knowledge of human interaction behavior, including communication, impression management, person perception, group dynamics and the emergence and development of conflicts and aggression is naturally required. In this regard specific legal knowledge about the etiology and prevalence of crime is also important. To perform police work successfully, police officers need psychological-diagnostic knowledge and knowledge about disorders, because police officers regularly come into contact with people suffering mental disorders or crisis situations. Since the police enforce official authority, police officers need to know the legal foundation in order to be able to apply their enforcement powers properly. This fundamental domain of knowledge also includes both criminal law and criminal procedure law in order to be able to investigate criminal offenses. Based on this, the police are authorized to use force under certain legal restrictions to enforce police interventions. On the one hand, this may require mental and physical skills for self-defense and the use of force. On the other hand, police officers must be capable of deploying police equipment and gear (such as firearms, distance electric impulse devices, batons, pepper spray) as a last resort. In addition, knowledge domains on the organizational and social context of the police play a central role. All knowledge domains presented form the basis for the process components.

### 3.3 Processing

The process components essentially relate to the mental processes of police officers. Therefore, we understand the present model as a cognitive action model with an emphasis on cognitions. As key processes we identify situational awareness including naturalistic decision-making, self-control and self-regulation competence including stress and emotion management as well as the police mindset in relation to self-reflection competence.

**Situational awareness and naturalistic decision-making**. Particularly during the first encounter, which begins with impression formation, and in the further course of the interaction, it is important that police officers are able to gain a solid understanding of the experience and behavior of the person involved, regardless of the presence of a mental disorder (Thielgen et al., 2022). Police officers need to rely on their situational awareness to be able to make appropriate decisions. Situational awareness coupled with naturalistic decision-making is described in the literature as a key competence of police officers for effective conflict management without the use of force and is therefore a major target of operational police training (Andersen and Gustafsberg, 2016; Andersen et al., 2015; Bennell et al., 2022; Blumberg et al., 2015; Dailey et al., 2024; Grier, 2015; Hansson and Borglund, 2024; Huhta, 2023; Huhta et al., 2021; Penney et al., 2022; Roberts and Cole, 2018; Stenshol et al., 2023; Koerner and Staller, 2022b; Wolfe et al., 2020).

According to Endsley (1995a,b, 2015a,b), situational awareness is a three-stage cognitive process of understanding. First, it involves the multimodal perception of as many relevant elements of the current situation as possible. Then the significance of the situational factors must be understood. Finally, a prediction is made about the further course of the situation (Endsley and Garland, 2000). On this basis, decisions are made between the mentally available options for action and finally concrete actions are carried out in the respective situation (Endsley, 2015a,b; Huhta, 2023).

With respect to human decision-making, a fundamental distinction is made between analytical and intuitive decision-making (Evans, 2008; Kahneman, 2011; Okoli and Watt, 2018; Strack and Deutsch, 2004). Analytical decision-making, also known as “thinking slow” or System 2 as referred to by Kahneman (2011), involves a thorough analysis of the situation and weighing up possible alternative courses of action. This process leads to precise results, but requires time and effort. Intuitive decision-making, also known as “thinking fast” or System 1 as referred to by Kahneman (2011), is based on repeated experience in relevant situations that require decisions and “gut feeling.” This process runs unconsciously and automatically. Therefore, it is particularly useful in dynamic and time-limited situations. On the one hand, intuitive decision-making is described as being subject to thinking errors and cognitive biases. On the other hand, this type of decision-making is seen as adaptive and functional in the context of naturalistic decision-making, as it enables decisions to be made under various constraints with high pressure and consequently to act functionally (Akinci and Sadler-Smith, 2020).

The concept of naturalistic decision making (NDM) provides a theoretical framework for decision-making behavior in naturally complex and dynamic environments such as police operations (Alpert and Rojek, 2011; Dailey et al., 2024; Hine et al., 2018; Klein, 2015; Klein, 2008). In such contexts, there is no possibility of analytical decision-making (Akinci and Sadler-Smith, 2020). Instead, experience in such situations provides knowledge in the form of schemas in associative long-term memory that mentally link situational stimulus patterns with options for action. If these stimulus configurations are recognized in natural situations, options for action can be accessed very quickly and appropriate actions can be taken (Klein et al., 2010). This allows police officers to remain capable of acting even in highly complex and dynamic operational situations with time pressure and stress (Dailey et al., 2024).

Research on police decision-making in naturalistic environments has shown that the quality of decisions made by experienced police officers is generally higher than those made by inexperienced police officers. For example, Suss and Ward (2018) found experience-based differences in police decision-making in complex, fast-moving and uncertain situations. Experienced police officers generated more relevant and less critical options for action. Huhta et al. (2023) showed that police officers with operational experience provide a more detailed description of the situation compared to those with no operational experience (see also Huhta et al., 2022; Stenshol et al., 2023). According to Boulton and Cole (2016), expertise plays a central role in the decision-making of police officers in armed confrontations. Police officers with operational expertise were able to react more flexibly to changing situations. Boulton and Cole (2016) understand this adaptive flexibility as a key feature of the expertise-based intuitive decision-making of police officers.

Behavioral observation is a prerequisite both for the assessment of individuals and for intuitive decision-making. To guide the assessment of a person in psychological distress or with psychopathological symptoms, we propose the use of so-called psychopathological measures as a heuristic screening tool (Ibrahim and Kattenberg, 2024). The descriptive psychopathological measures are based on general, relatively objectively observable categories of experience and behavior, such as delusions (Saß and Hoff, 2016; cf. AMDP system, Arbeitsgemeinschaft für Methodik und Dokumentation in der Psychiatrie AMDP, 2023). Abnormalities in these categories provide information about the acute mental state of the person concerned.

Similar to the recording of a psychopathological measures, police officers can register apparently remarkable symptoms that are relevant in terms of danger to themselves and others and use them as a basis for their communication without making etiological assumptions. However, a mental disorder or a conspicuous symptom can only be one risk factor. The first step is to acknowledge inter-individual differences in people’s perception and behavior. It is crucial to accept that interaction partners may behave differently than expected, which means being able to anticipate different situations and courses of action. In addition, possible conflict constellations and situational dynamics, such as alcoholization, heat, groups, partnership separation, etc., or the interaction behavior of both sides, including categorization, stereotyping and cognitive biases (e.g., authoritarian, unfriendly or discriminatory behavior of police officers) can also have a significant influence on the interactional course of an operational situation. It is fundamentally important to create police officers’ awareness of the range of human experience and behavior as well as acceptance of what is possible and different (possibly incomprehensible). It is fundamentally important to create police officers’ awareness of the wide range of human perception and behavior as well as acceptance of what is possible and different (perhaps even incomprehensible).

Scenario-based operational training is about repeating situation variations in such a way that, on the basis of situational awareness in terms of pattern recognition, action schemas comprising action sequences and action effects of a non-violent solution to the situation can be learnt and easily recalled in order to make non-violence more likely as a result (Klein, 1989, 1993, 1997, 2008, 2015; Klein et al., 2010). Operational training that focuses on situational awareness and decision-making generally shows improvements in performance (Andersen and Gustafsberg, 2016; Andersen et al., 2018; DiNota and Huhta, 2019; Hansson and Borglund, 2024; Nieuwenhuys and Oudejans, 2011; O’Hare and Beer, 2020; Olma et al., 2024).

**Self-regulation, stress regulation and emotion regulation**. Self-regulation (also referred to as self-control or action control) is a generic term for all conscious and unconscious mental processes that enable people to pursue their goal-oriented actions (Karoly, 1993; Vohs and Baumeister, 2017). According to Carver and Scheier (1998), self-control is the adaptation of actions by comparing current actions with a desired target state. In the case of self-observed deviations, actions are adapted in order to achieve the target state. In Baumeister et al.'s (2007) resource model of self-control, self-control is an ability of limited capacity called “willpower.” Similar to a muscle, self-exhaustion can occur through use and self-regulation can decrease (Baumeister and Vohs, 2007; Muraven and Baumeister, 2000). For example, Staller et al. (2019) showed that police officer candidates were more likely to use force against provocative citizens in a video-based exercise if their self-control had previously been exhausted (Staller et al., 2018). Donner et al. (2017) found that police officers with low self-control were more likely to be involved in shootings. Overall, the lack of self-control seems to play a central role in the context of deviant and undesirable behavior of police officers (Donner, 2020; Donner et al., 2018; Donner et al., 2016; Donner and Jennings, 2014). In this perspective, low self-control plays the role of a criminogenic risk factor that prevents the inhibition of aggressive impulses to act (Denson et al., 2012). Conversely, a high level of self-control helps to prevent the occurrence of aggression (Denissen et al., 2018; DeWall et al., 2011). Self-control can also relate to emotional or stressful states (Gross, 2002; Gross, 1998). Since stress experiences generally have a negative impact on information processing and action planning and execution, self-regulation of stress and training in this area play an important role in maintaining the ability to act under stressful conditions in the field (Baldwin et al., 2022; Keech et al., 2020; Nieuwenhuys et al., 2015; Nieuwenhuys et al., 2012). The adaptation of emotions and stress reactions, for example through attention control or re-appraisal, can minimize handling errors, for example when shooting (Andersen et al., 2018; Vickers and Lewinski, 2012). Research on police officers shows that police officers are prevented from suffering stress-related performance losses if they have received scenario training under high stress conditions that is representative of an operational situation (Dailey et al., 2024; DiNota and Huhta, 2019; Nieuwenhuys and Oudejans, 2010, 2011; Oudejans, 2008; Oudejans and Pijpers, 2009).

**Self-reflection and mindset**. The basic police attitude is one of the internal psychological prerequisites for police action (Staller et al., 2022c). These basic attitudes of police officers have so far been studied primarily in the context of police organizational culture (Ingram et al., 2018; Paoline III et al., 2021). Recently, the basic attitudes of police officers have been discussed with regard to the “warrior-guardian” dichotomy and its influence on police action (Boe et al., 2020; Carlson, 2020; Clifton et al., 2021; Koslicki, 2020; Kurtz and Colburn, 2019; Li et al., 2021; McLean et al., 2019b; Rahr and Rice, 2015; Schuck, 2024; Smith et al., 2020; Stoughton, 2015, 2016). This attitudinal concept refers to the way in which police officers perceive their own professional role on duty and what typical patterns of action are accepted by them. Police officers with a “warrior mentality” perceive people and the world around them as dangerous and see themselves as crime fighters, while police officers adopting a “guardian mentality” seek to serve and protect the community by working with the public in a spirit of trust (McLean et al., 2020; Stoughton, 2015).

At the behavioral level, these attitudes appear to correlate with certain interpersonal behavior patterns (Schade and Thielgen, 2023). Whether and to what extent de-escalating communication, trusting cooperation in the sense of procedural justice or even the use of force occurs during police operations can therefore depend on the individual professional attitude of the police officer. McLean et al. (McLean et al., 2019b; McLean et al., 2019a, p. 1113) provide empirical findings confirming that “guardian officers are less likely (and warrior officers are more likely) to use force when unnecessary or inappropriate.” However, a systematic investigation of the empirical relationships between the attitudes of police officers and their action in police operations has not yet been conducted (Paoline III et al., 2021). With regard to individuals in exceptional and crisis situations and/or with mental disorders, the question arises as to how the perception of needs, the assessment of dangerousness, the legitimization of the use of force and stigmatizing attitudes toward mentally ill people influence police officers’ actions in duty (Mengual-Pujante et al., 2022).

Police officers may be more or less aware of their own basic assumptions about people in general, people with mental health problems or disorders in particular, the nature of human interaction and communication, conflict and crisis dynamics as well as the police self-image, the relationship to the public and the professional role. Staller et al. (2022b) understand reflexivity as part of an experience-driven learning process and present it as a core feature of professional police work (Schön, 1983; Staller et al., 2022d; Wood and Williams, 2017). Its importance for police work is clarified by Christopher (2015, p. 328, cited in Staller et al., 2022b, p. 42): “It is crucial that members of the police profession learn to think critically, conceptually and creatively when confronted with situations that require analysis and when developing solutions to problems. They also need the ability to learn from their experiences.” [„Es ist entscheidend, dass die Angehörigen der Polizeiberufe lernen, kritisch, konzeptionell und kreativ zu denken, wenn sie mit Situationen konfrontiert werden, die einer Analyse bedürfen, und wenn sie Problemlösungen entwickeln. Sie brauchen auch die Fähigkeit, aus ihren Erfahrungen zu lernen“]. The aim of this critical self-reflection is to identify the underlying assumptions that guide one’s own actions and to be able to adapt them accordingly.

### 3.4 Action

Zaiser et al. (2022, p. 258) describe “police communication skills and de-escalative action skills” as “key competencies that are superior to the use of other means in contact with citizens and underpin police action as a whole.” [„Schlüsselkompetenzen, die dem Einsatz anderer Mittel im Bürgerkontakt übergeordnet sind und das polizeiliche Handeln als Gesamtes tragen.“] (Wang et al., 2024). As already mentioned with respect to the concept of ability and competence, in professional contexts it is about context-specific performance and its observable execution for coping with practical requirements. The action components of the GIMP relate to specific actions of police officers in the field. Based on an understanding of the concrete situational dynamics, including their own logic of thought and action as well as that of their interaction partners, and an assessment of the physical and psychological level of functioning and the resulting risk constellations, we propose a structuring of situational communication behavior with the help of five action dimensions: (a) impression regulation, (b) distance regulation, (c) time regulation, (d) language regulation, (e) relationship regulation. The interplay of these five regulatory dimensions should enable flexible adaptation to the interaction partners. The aim of the intervention is not to apply use of force (Staller et al., 2022a; Staller et al., 2021; Wolfe et al., 2020; Zaiser et al., 2022;).

Active listening developed by Gordon (1975) and rooted in Rogers (1951, 1959) is a non-coercive communication technique that positively affect interactions and communication outcomes (Jones et al., 2019; Simon, 2018; Weger et al., 2014) by avoiding typical human responses such as evaluating, judging, advising or moralizing and instead emphasizing interest, empathy and reflection (Rogers and Farson, 1987). As in other fields of application (e.g., Hoppe, 2007), practitioners and researchers in the police context have identified active listening as an important communication skill during initial interactions of crisis situations. The use of active listening in the initial stages of negotiation is seen as a critical factor in resolving the crisis, making it an essential skill for any (hostage) negotiation (Royce, 2005; Weger et al., 2014). Indeed, Johnson et al. (2017) found that crisis (hostage) negotiators of police agencies list active listening at the top of their qualities required. The acronym MOREPIES (Minimal Encouragers, Open-Ended Questions, Reflecting, Emotion Labeling, Paraphrasing, I-Messages, Effective Pauses, Summary) was developed by the US Federal Bureau of Investigation (FBI) to remind negotiators the active listening skills while under pressure (Zaiser and Staller, 2015). Within the revised Behavioral Influence Stairway Model (BISM; Ireland and Vecchi, 2009; Vecchi et al., 2019; Wong et al., 2021), which can be applied to various crisis situations, for example that of suicidal individuals, active listening skills are the foundation of a relationship building process between negotiator and the interlocutors. The model assumes that several stages of relationship building must be passed through in sequential order to convince a person to change their behavior. For example, refraining from a planned suicide only occurs if different stages have been successfully completed and the negotiator is credible to the person in crisis. First, it is necessary to find an empathic access by understanding the situation of the other person in order to develop a relationship named rapport that makes it possible to influence the person in crisis (Vecchi, 2022; Wong et al., 2021).

## 4 Concluding remarks

The main components of the GIMP can be categorized as (1) knowledge components, (2) process components and (3) action components. The knowledge components provide the knowledge required for police work from psychology, law, criminology, criminalistics, police gear and equipment preparation as well as police self-defense and use of force, taking into account the personal characteristics (KSOAs) necessary for the successful mastery of police operations. The central process components of the GIMP concern the individual cognitive factors of the acting police officers. In particular, self-regulatory skills with regard to individual stress and emotion management (e.g., Berking et al., 2010; Schaible and Six, 2016; Sweeney, 2022), the individual mindset in conjunction with reflection skills (e.g., Staller et al., 2022b) as well as situational awareness and naturalistic decision-making (e.g., Huhta et al., 2023; Koerner and Staller, 2022b) can be regarded as key factors for coping with police operations. The action components refer to specific action steps that the police officer takes toward the interaction partner. In addition to the acute conflict and crisis dynamics and the specific interaction behavior including any psychopathological symptoms, police officers must assess the general level of functioning and the resulting danger posed by the interaction partners to themselves and others. In terms of adaptive action flexibility, police officers can modulate their actions in terms of impression, relationship, language, distance and time in order to carry out a police action. According to the revised BISM (Vecchi et al., 2019), active listening skills should enable an empathic access to the interaction partner to create a sustainable working relationship (rapport), on the basis of which influence can ultimately be exerted on the interaction partner in order to achieve a behavioral change (e.g., to calm down, to put the knife away, to stop assaulting).

Police conduct regularly takes center stage when police operations fail and alleged police violence, i.e., the disproportionate and unjustified use of force by police officers against citizens, becomes public. Then, the search for explanations is understandable and should be taken seriously. However, effective police action is not only important following high-profile media reports of negative police incidents. Recently, the question of effective or professional police action in Germany has been discussed from various perspectives, particularly in academia (e.g., Hunold and Singelnstein, 2022; Hunold and Ruch, 2020; Küppers, 2022; Schade and Thielgen, 2023; Staller and Koerner, 2022; Staller et al., 2023e; Staller et al., 2023d; Staller et al., 2023c; Staller et al., 2023b; Staller et al., 2023a; Zum-Bruch, 2019). There are currently some considerations on the issue of professional operational action by police officers: “Gewaltreduzierendes Einsatzmodell GeredE” (Staller et al., 2022a), “Einsatzmodell für aggressiven Verhaltensweisen im Kontext psychischer Störungen” (Biedermann and Ellrich, 2022), “Modell der Einsatzkompetenz” (Schmalzl, 2022), “Modell Einsatzkompetenz 4.0” (Körber, 2020), “KODIAK” (Lorei et al., 2024). However, a general, empirically tested theory of action for police operations does not yet exist. On the one hand, the individual factors presented in the GIMP are theoretically relevant and plausible. On the other hand, there is empirical evidence for the significance of the cognitive components presented. The GIMP primarily relates to the area of police patrol duty. A transfer to other areas of police work (e.g., criminal investigation department, special units) is conceivable. In connection with the explanation of the perception and behavior of police officers in the field with the help of models such as the one presented here, it should be noted that there is generally a danger of monocausally explaining concrete characteristics of perception and behavior that have arisen in a complex set of conditions. One factor alone cannot fully explain police actions (Lee, 2023).

The present article is methodically based on a narrative literature review. Due to its non-systematic nature, certain methodological limitations are inherent (Grant and Booth, 2009). Firstly, there is an increased risk of selection bias, as the studies included were not selected according to strictly standardized and therefore reproducible inclusion and exclusion criteria. For example, the PRISMA (Preferred Reporting Items for Systematic Reviews and Meta-Analyses) is an established and widely used instrument, consisting of a checklist and a flowchart, to improve the transparent and complete report of systematic literature reviews and meta-analyses (Page et al., 2021). Secondly, there is no formalized quality check of the sources included, as provided by standardized evaluation instruments in systematic reviews, for example using the Critical Appraisal Skills Program (CASP) checklists (Critical Appraisal Skills Programme (CASP), 2018; Long et al., 2020). Thirdly, it should be noted that narrative reviews do not only include quantitative studies. Rather, they are not intended to provide a quantitative synthesis of empirical evidence, as is the case with meta-analyses (Borenstein et al., 2021; Higgins et al., 2022). Single results were not pooled in a statistically robust analysis. Nor is any other statistical analysis conducted. Consequently, differences between the included studies in terms of effect size, sample type and size, or data collection methods remain unweighted, which makes a systematic evaluation of the overall evidence difficult or impossible. Therefore, conclusions are derived from subjective interpretations rather than aggregated data, which significantly limits the transferability and generalizability of results to other practical contexts or target populations. However, it should be acknowledged that even systematic literature reviews and meta-analyses are not immune to bias (for a critical evaluation of systematic reviews and meta-analyses, see Ioannidis, 2016; Egger et al., 1997).

Despite these limitations, the narrative approach of a literature review allows a flexible and context-sensitive examination of the state of research, especially in heterogeneous or as yet little systematically investigated research areas. In this sense, a narrative literature review can help to identify research gaps and future research directions. The proposed model is intended to create a theoretical framework focusing on the process components. It attempts to characterize those cognitive processes of police officers underlying police actions that are relevant to the successful accomplishment of police operations. It also highlights the prerequisites for the shaping of cognitive processes and the resulting police actions in terms of learning or educational content. However, these knowledge components required for the expression and development of cognitive processes may not be adequately described here. There is also no conclusive and exhaustive empirical validation. Future research needs to reveal empirically established.

Future research needs to focus on the question of how and with what content police officers should be trained. On the basis of the GIMP, both content and methodology of police education and training can be derived and designed along the three components (knowledge, processes, action) of the GIMP aimed to establish the essential skills of police officers for successfully coping with real operational requirements. In terms of the constructive alignment model (Biggs, 1996, 1999), the learning outcomes (action components as target competencies required for the successful fulfillment of police operations), the teaching and learning activities (knowledge components as contents and methods taught and applied in police education and training) and the assessment of training performance (as a predictor of future job performance) should be aligned with each other (Biggs and Tang, 2011; Gallagher, 2017). The process components mediate between the knowledge and action components. Future research also needs to operationalize the action components and to test them empirically with regard to the operational success. The objective should be to identify empirically tested behavioral patterns of police officers. Finally, both content and methods of police education and training can be subjected to empirical evaluation in terms of skills acquisition.

With his famous bon mot “All models are wrong, some are useful,” the British statistician Box (1976) makes it clear that scientific (including mathematical) models should have a certain usefulness for everyday life rather than claiming to be true. In this respect, police operational models can also be understood as a theoretical framework for relevant conditional structures without sufficiently explaining operational phenomena or establishing causality. The GIMP can provide a theoretical foundation for police research, education and training. Furthermore, it can stimulate the formulation of hypotheses for empirical testing and lay the foundation for an evidence-based evaluation of education and training measures. In this way, a “what works” approach can be rigorously pursued.

## Data Availability

The original contributions presented in the study are included in the article/supplementary material, further inquiries can be directed to the corresponding author.

## Author’s note

The article is an extended version of Schade, S. & Thielgen, M. M. (in press). Ein Allgemeines Einsatzmodell [A General Intervention Model of Policing]. In M. S. Staller, S. Koerner & B. Zaiser (Eds.), *Polizei und Menschen in psychischen Krisen* [The police and individuals with mental health crisis]. Springer Gabler.
